# Overexpression of Glucocorticoid Receptor β Enhances Myogenesis and Reduces Catabolic Gene Expression

**DOI:** 10.3390/ijms17020232

**Published:** 2016-02-11

**Authors:** Terry D. Hinds, Bailey Peck, Evan Shek, Steven Stroup, Jennifer Hinson, Susan Arthur, Joseph S. Marino

**Affiliations:** 1 Center for Hypertension and Personalized Medicine, Department of Physiology and Pharmacology, University of Toledo College of Medicine, Toledo, OH 43614, USA; terry.hinds@utoledo.edu; 2 Laboratory of Systems Physiology, Department of Kinesiology, University of North Carolina Charlotte, Charlotte, NC 28223, USA; bpeck3@uncc.edu (B.P.); eshek@uncc.edu (E.S.); sstroup4@uncc.edu (S.S.); jhinso47@uncc.edu (J.H.); sarthur8@uncc.edu (S.A.)

**Keywords:** glucocorticoid receptor α 2, glucocorticoid receptor β 3, GRβ 4, GRα 5, atrophy 6, MAFbx 7, MuRF1 8, dexamethasone 9, myogenesis

## Abstract

Unlike the glucocorticoid receptor α (GRα), GR β (GRβ) has a truncated ligand-binding domain that prevents glucocorticoid binding, implicating GRα as the mediator of glucocorticoid-induced skeletal muscle loss. Because GRβ causes glucocorticoid resistance, targeting GRβ may be beneficial in impairing muscle loss as a result of GRα activity. The purpose of this study was to determine how the overexpression of GRβ affects myotube formation and dexamethasone (Dex) responsiveness. We measured GR isoform expression in C_2_C_12_ muscle cells in response to Dex and insulin, and through four days of myotube formation. Next, lentiviral-mediated overexpression of GRβ in C_2_C_12_ was performed, and these cells were characterized for cell fusion and myotube formation, as well as sensitivity to Dex via the expression of ubiquitin ligases. GRβ overexpression increased mRNA levels of muscle regulatory factors and enhanced proliferation in myoblasts. GRβ overexpressing myotubes had an increased fusion index. Myotubes overexpressing GRβ had lower forkhead box O3 (Foxo3a) mRNA levels and a blunted muscle atrophy F-box/Atrogen-1 (MAFbx) and muscle ring finger 1 (MuRF1) response to Dex. We showed that GRβ may serve as a pharmacological target for skeletal muscle growth and protection from glucocorticoid-induced catabolic signaling. Increasing GRβ levels in skeletal muscle may cause a state of glucocorticoid resistance, stabilizing muscle mass during exposure to high doses of glucocorticoids.

## 1. Introduction

Chronic glucocorticoid (GC) treatment and prolonged elevations of endogenous GC production cause skeletal muscle atrophy and reduce the adaptive response of skeletal muscle to injurious and atrophic events [1,2,3,4]. For example, muscle atrophy is evident both in patients with Cushing’s syndrome, diabetes, and renal disease in whom GC levels are endogenously elevated, and in patients with chronic obstructive pulmonary disease, cancer, and chronic inflammatory disease for whom chronic GC therapy is a part of treatment [5]. GCs activate the glucocorticoid receptor (GR), a hormone activated transcription factor [6,7,8]. Due to alternative splicing of a single gene, there are two major GR isoforms: GRα and GRβ [9]. GRβ has a truncated GC ligand-binding domain, which prevents GC binding and is a dominant negative inhibitor of GRα [9,10,11].

While the physiological roles of GRβ are not completely understood, increased GRβ expression has been linked to GC resistance in patients suffering from severe asthma [12,13,14,15], leukemia [16], cancer [17], and inflammation [18], which reduces the therapeutic potential of GCs. Additionally, transcriptome analysis of cultured cells overexpressing GRβ indicated intrinsic transcriptional activities independent of GRα [19]. We recently demonstrated that GRβ positively regulates cell proliferation by attenuating phosphatase and tensin homolog deleted on chromosome 10 (PTEN) expression and by increasing Akt1 phosphorylation in 3T3-L1 cells [20]. Akt1 regulates embryonic and fetal growth, which suggests that GRβ may have a predominant role in development and proliferation. Recently, GRβ has been shown to regulate the growth of glioblastoma [21] and prostate cancer cells [22] as well.

Proliferation and differentiation of skeletal muscle myocytes are necessary for the cellular and molecular events that orchestrate skeletal muscle repair, adaptations to inactivity or exercise, as well as the basal maintenance of skeletal muscle size [23,24]. Further, Akt and PTEN, which are regulated by GRβ in 3T3-L1, glioblastoma, and prostate cancer cells, also contribute to the proliferation and differentiation of skeletal muscle cells. Knockdown of Akt1 expression using shRNA markedly reduced MyoD and myogenin protein expression in differentiating myocytes [25]. Additionally, myotube formation was abolished in the absence of Akt1, but not Akt2 [25]. PTEN knockdown in rat myoblasts increased myosin heavy chain expression threefold in early stage myotubes and nearly doubled the differentiation rate [26]. Unlike that of 3T3-L1, glioblastoma, and prostate cancer cells, the contribution of GRβ to the regulation of factors involved in the skeletal muscle myogenic program, such as MyoD and myogenin, remains to be determined.

It is well known that GCs induce muscle atrophy. The binding of GCs to the ligand-binding domain of GRα causes translocation to the nucleus and binding to glucocorticoid response elements (GREs) in the promoter region of genes. Specifically, GRα binds to GREs in the promoter of forkhead box O (Foxo) transcription factors and enhances expression [27]. This results in a Foxo-dependent increase in muscle atrophy F-box/Atrogen-1 (MAFbx) and muscle ring finger 1 (MuRF1), E3 ubiquitin ligases necessary for GC-induced muscle myopathy [28,29,30,31,32,33,34,35]; suppression of MAFbx and MuRF1 inhibits GC-induced protein degradation [36]. Despite our growing knowledge surrounding the functions of GRα in the regulation of skeletal muscle atrophy, very little is known about the contribution of GRβ to these events. The ability of GRβ to inhibit GRα suggests that it may be an inhibitor of atrophic signaling causing a state of GC resistance in skeletal muscle. The overall purpose of this study was to determine if overexpression of GRβ in C_2_C_12_ muscle cells alters myotube formation and sensitivity to exogenous GC.

## 2. Results

### 2.1. GRβ Responsiveness to Dexamethasone and Insulin

**Figure 1:**
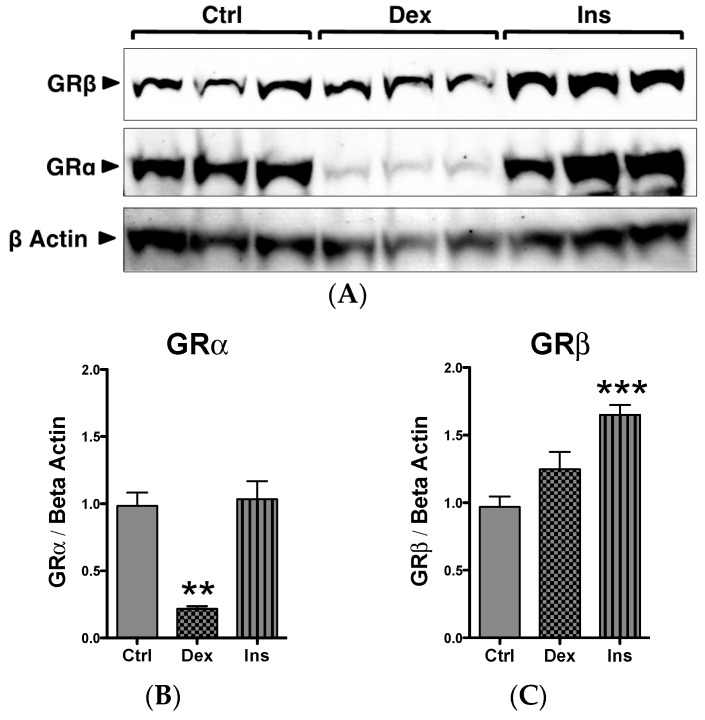
GR expression and responsiveness in C_2_C_12_ myoblasts. (**A**) Western blot of C_2_C_12_ myoblasts treated with vehicle (Ctrl), dexamethasone (Dex), or insulin (Ins) for 24 h; (**B**) Quantification of GRα protein expression in response to Dex and Ins; ** *p* < 0.01 compared to Ctrl; (**C**) Quantification of GRβ protein expression in response to Dex and Ins; *** *p* < 0.001 compared to Ctrl. *n* = 3 experiments. Data expressed as mean ± SEM.

The roles of GRα and GRβ in the skeletal muscle myogenic program are unknown. We have previously shown that the mouse muscle cell line C_2_C_12_ expresses both GRα and GRβ [20]. In Figure 1A,B, we show that the C_2_C_12_ myoblasts respond to the GC dexamethasone (Dex), causing a significant (*p* < 0.01) suppression of GRα and no change in GRβ expression. We have previously reported in mouse embryonic fibroblast (MEF) that Dex decreased GRα as part of a negative feedback loop [9]. However, we also showed that MEF cells exposed to Dex had increased GRβ expression, which is a known inhibitor to GRα, and was potentially a part of the negative feedback loop. The mechanism in myocytes may potentially be different for the long-term negative feedback of GCs. The response of GRβ to GCs is consistent with previous findings in human skeletal muscle myoblasts and myotubes [37]. We also showed in the MEF cells that GRβ mRNA [9] and protein [20] increased in response to insulin. In the present study, we also show that insulin significantly (*p* < 0.001) increased GRβ protein expression (Figure 1C) in C_2_C_12_ myoblasts, with no effect on GRα (Figure 1B).

### 2.2. GR Isoform and Muscle Regulatory Factor mRNA Levels through Differentiation

**Figure 2:**
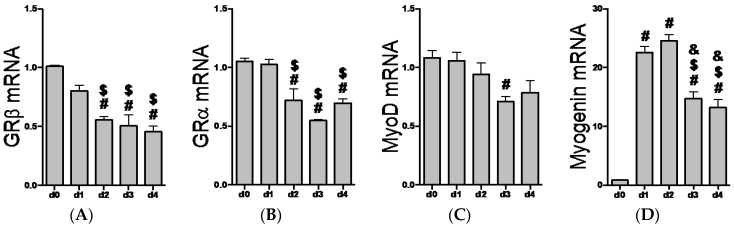
Changes in glucocorticoid receptor and myogenic mRNA expression during myotube formation. C_2_C_12_ myoblasts were induced to differentiate into myotubes starting at ~90% confluence, d0 (day zero). Differentiation was carried out for four days, d1 (day one) through d4 (day four). (**A**) GRβ. # *p* ≤ 0.0009 compared to d0; ^$^ *p* < 0.05 compared to d1; (**B**) GRα. ^#^ *p* < 0.01 compared to d0; ^$^ *p* < 0.01 compared to d1; (**C**) MyoD. ^#^ *p* = 0.0179 compared to d0; (**D**) Myogenin. ^#^ *p* < 0.0001 compared to d0, ^$^ *p* < 0.0001 compared to d1, and ^&^ *p* < 0.0001 compared to d2 (day two). *n* = 3 to 6 experiments per time point. Data expressed as mean relative quantification (RQ) ± SEM.

We recently reported the expression of GRβ in C_2_C_12_ myoblasts [20], while others have identified GRβ mRNA in human myoblasts and myotubes [37]. However, it is currently unknown how the expression pattern of GR isoforms changes through the myogenic program. Interestingly, GRβ and GRα mRNA levels decrease similarly when transitioning from myoblasts to myotubes (Figure 2A,B). As expected, MyoD mRNA levels gradually decline through differentiation (Figure 2C), while myogenin transcript levels show a significant (*p* < 0.0001) increase beginning one day into the differentiation process (Figure 2D). In an unchallenged and basal state, these data indicate that both GR isoforms follow the same temporal pattern of expression during myotube formation.

### 2.3. Overexpression of GRβ Increases Muscle Regulatory Factor mRNA Levels

**Figure 3:**
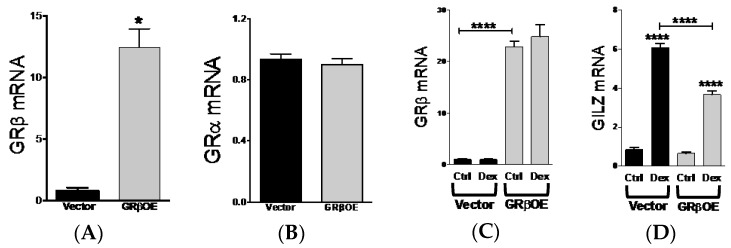
GRβ overexpression reduces dexamethasone responsiveness in C_2_C_12_ myoblasts. (**A**) Myoblasts overexpressing GRβ (GRβOE) had a significant increase in GRβ mRNA expression compared to vector transfected cells. * *p* = 0.016; (**B**) GRα mRNA expression was not altered by GRβ overexpression; (**C**) GRβ mRNA expression was not altered in GRβOE cells following 2-h dexamethasone (Dex) treatment. **** *p* < 0.0001 as indicated by brackets; (**D**) Glucocorticoid-induced leucine zipper (GILZ) mRNA levels showed an abrogated response to Dex in GRβOE myoblasts. **** *p* < 0.0001 compared to respective Ctrl and as indicated by brackets. *n* = 3 to 5 experiments. Data expressed as mean RQ ± SEM.

The ability for GRβ to inhibit the activity of GRα makes it an attractive target to blunt the side effects typically associated with GC treatment, particularly regarding the maintenance of skeletal muscle mass. Therefore, we overexpressed mouse GRβ cDNA in C_2_C_12_ cells (GRβOE) by lentivirus and determined how elevated GRβ expression affected GC responsiveness and mRNA levels of MyoD and myogenin. GRβOE myoblasts had approximately 12.5-fold higher GRβ expression compared to vector cells (Figure 3A), while GRα mRNA expression was not altered (Figure 3B). Consistent with GRβ responsiveness to Dex in C_2_C_12_ myoblasts (Figure 1), GRβ mRNA levels were not influenced by Dex in GRβOE cells (Figure 3C). Furthermore, Dex responsiveness of glucocorticoid-induced leucine zipper (GILZ), a target of GRα, was significantly reduced in GRβOE myoblasts (Figure 3D), suggesting reduced GRα activity with elevated GRβ.

**Figure 4:**
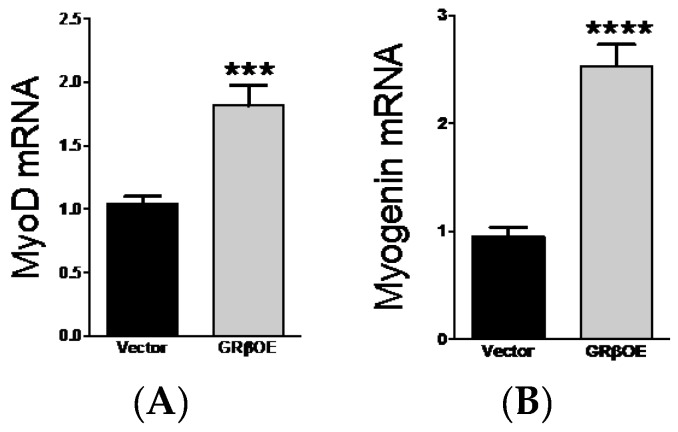

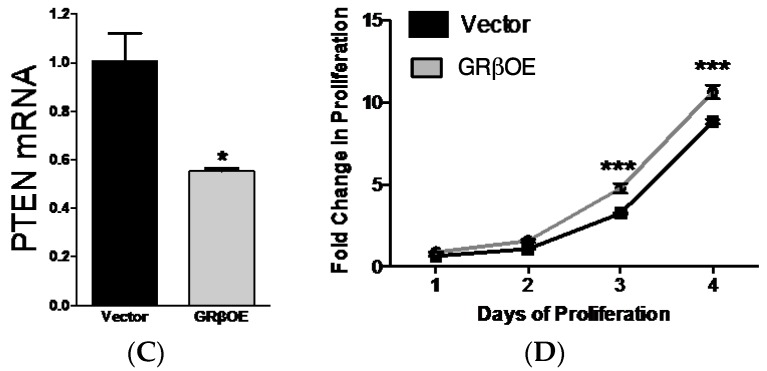
GRβ overexpression increases myogenic mRNA expression and proliferation. (**A**) MyoD and (**B**) Myogenin mRNA levels were significantly elevated in GRβOE myoblasts compared to vector. *** *p* = 0.0008, **** *p* < 0.0001; (**C**) PTEN mRNA levels were suppressed in GRβOE myoblasts. * *p* = 0.017. *n* = 3 to 6 experiments; (**D**) An MTT proliferation assay showed that GRβOE myoblast had an increase in proliferation at days 3 and 4 of assessment compared to vector. *** *p* = 0.0008 at day 3 and *p* = 0.0001 at day 4 between cells types and the time point indicated. *n* = 3 experiments per time point. Data expressed as mean RQ ± SEM for real time PCR analysis and mean fold change ± SEM for proliferation.

Myogenin and MyoD are important to muscle regulatory factors that regulated the progression from myoblasts to multinucleated myotubes. In ~90% confluent cultures, myogenin mRNA was approximately 2.5-fold higher (Figure 4A) and MyoD mRNA 1.75-fold higher (Figure 4B) in GRβOE compared to vector cells. Consistent with our previous findings [20], overexpression of GRβ mitigated the expression of the tumor suppressor, PTEN (Figure 4C), which suggests that proliferation would be enhanced. As determined by an MTT (3-(4,5-dimethylthiazol-2-yl)-2,5-dipheyltetrazoline bromide) assay over a four-day period, GRβOE cells showed a significant enhancement of proliferation on days 3 and 4 (Figure 4D). These data suggests that GRβ may contribute to the regulation of MyoD and myogenin expression, which could enhance proliferation, myonuclear fusion, and myotube formation.

### 2.4. Overexpression of GRβ Enhances the Myotube Formation

**Figure 5:**
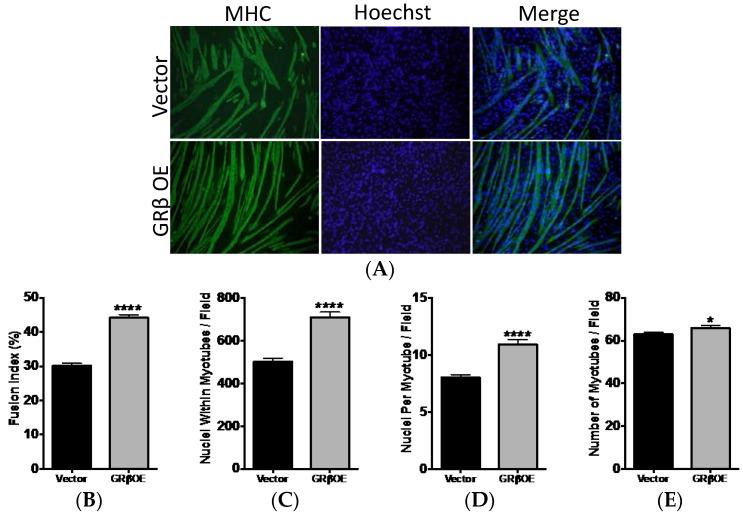
GRβ overexpression enhances fusion and myotube formation. (**A**) Myoblast fusion and myotube formation was assessed in vector and GRβOE cells using myosin heavy chain (MHC) labeling following four days of differentiation. Myotubes were identified as MHC+ cells with a minimum of two nuclei; (**B**) The fusion index; (**C**) total nuclei within myotubes; (**D**) nuclei per myotube; (**E**) the number of myotubes, were all significantly greater in GRβOE cells. * *p* < 0.05, and **** *p* < 0.0001 compared to vector. *n* = 6 to 9 experiments. 5 to 7 fields were analyzed per experiment by blinded investigators. All images analyzed at 10× magnification. Data expressed as mean ± SEM.

To determine whether overexpression of GRβ altered myotube formation, we differentiated GRβOE and vector myoblasts for four days and labeled myotubes with sarcomeric myosin heavy chain (MHC) (Figure 5A). A myotube was determined as a MHC positive (MHC+) cell with two or more nuclei. The fusion index was significantly (*p* < 0.0001) higher in GRβOE myotubes (Figure 5B). Moreover, the number of nuclei within myotubes (Figure 5C), the number of nuclei per myotube (Figure 5D), and the total number or myotubes (Figure 5E) were all increased in GRβOE compared to vector cultures.

### 2.5. Overexpression of GRβ Blunts Dex-Induced Catabolic Gene Expression

**Figure 6:**
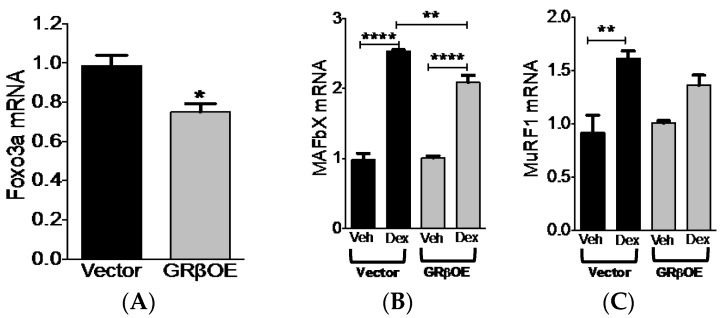
Dexamethasone-induced atrogene expression is reduced in GRβOE myotubes. (**A**) Foxo3a mRNA was reduced in GRβOE myotubes. * *p* = 0.025; (**B**) Muscle atrophy F-box (MAFbx; also known as atrogen-1); (**C**) muscle ring finger 1 (MuRF1) mRNA response to 24 h of Dex exposure was diminished in GRβOE myotubes. ** *p* < 0.01 and **** *p* < 0.0001. *n* = 3 experiments. Data expressed as mean RQ ± SEM.

GRα-mediated activation of the Foxo-atrogene (MAFbx and MuRF1) pathway is a well established mediator of muscle atrophy. Due to the ability of GRβ to inhibit GRα activity, we tested whether Dex-induced MAFbx and MuRF1 mRNA expression were abrogated in GRβOE myotubes. First, we measured mRNA levels of Foxo3a, a transcription factor known to regulate atrogene expression [28]. Foxo3a transcript levels were significantly (*p* = 0.025) reduced in GRβOE myotubes (Figure 6A). As expected, Dex treatment caused an increase in MAFbx and MuRF1 mRNA levels in vector myotubes (Figure 6B,C). MAFbx mRNA levels increased in GRβOE myotubes in response to dexamethasone. However, this response was significantly (*p* = 0.0091) blunted compared to vector myotubes (Figure 6B). Although dexamethasone caused a small rise in MuRF1 mRNA in GRβOE myotubes, this was not significant (*p* = 0.1443) (Figure 6C). While MuRF1 mRNA levels were approximately 18% lower in GRβOE cells compared to vector cells treated with Dex, this was not found to be statistically significant (*p* = 0.3678). Taken together, Dex-induced expression of atrogenes, particularly that of MAFbx, is reduced with overexpression of GRβ, providing a modest level of protection against the deleterious effects of GC exposure.

## 3. Discussion

The detection of GRβ in mouse [9] has provided a new model to investigate whether GRβ contributes to GC sensitivity and GR-mediated skeletal muscle atrogene (MAFbx and MuRF1) expression. In this investigation, we showed for the first time that overexpression of GRβ enhances myotube formation and reduces GC responsiveness in C_2_C_12_ mouse muscle cells. We demonstrated that GRβ protein levels in C_2_C_12_ mouse muscle cells do not change following GC exposure, consistent with findings in human skeletal muscle cells [37]. Furthermore, GRβ protein expression increased in response to 24 h of insulin exposure. Insulin is a mitogenic factor that regulates metabolism, cell growth, and protein balance. Insulin resistance, a known side effect of GC therapy, contributes to muscle atrophy via reduced protein synthesis and increased protein degradation by genomic and non-genomic interference with several kinases in the insulin-signaling pathway [29,38,39,40,41,42,43].

Rats exposed to cortisone for five days had reduced insulin receptor phosphorylation and reduced insulin receptor substrate-1 (IRS1) content [44]. Dex-induced GR activation caused a significant reduction in IRS1/phosphoinositide 3-kinase (PI3K) association, attributed to an increase in GR/PI3K interaction, which was attenuated in mice lacking GR in skeletal muscle [45]. More recently, a glucocorticoid response element was found in the promoter region of p85α (regulatory subunit of PI3K) in C_2_C_12_ myotubes [46]. Overexpression of p85α caused a GC-like effect, including a reduction in myotube size, while reducing p85α expression protected myotubes against GC-induced suppression of insulin signaling [46]. Importantly, we showed that overexpression of GRβ enhanced insulin-stimulated Akt phosphorylation in MEF and 3T3 cells [20]. Our data reported here, together with prior findings, underscore multiple mechanisms by which GCs can induce insulin resistance via activation of GRα across genomic and non-genomic regulation. Therefore, it is intriguing to postulate that GRβ has the potential to combat GRα-mediated insulin resistance in skeletal muscle via multiple routes; GRβ/GRα heterodimers may prevent genomic and non-genomic GRα interactions, and GRβ may enhance insulin signaling downstream of the proximal kinases most affected by GRα activation. Enhanced GRβ expression in this regard, may reduce GRα activity under conditions of GC exposure, which may help preserve muscle mass. Indeed, further characterization *in vivo* is required before drawing significant conclusions. However, these data do allude to a mitogen-sensitive pathway for GRβ in skeletal muscle.

Cell proliferation is a tightly regulated process interconnected with mitogen-sensitive pathways, particular kinases that are insulin-responsive. PTEN is a known regulator of cellular growth, and in our prior work we have shown that overexpression of GRβ enhances proliferation of 3T3 cells via inhibition of PTEN and increased phosphorylation of Akt [20], corroborating results from tumor studies [21,22]. PTEN also plays a prominent role in the skeletal muscle myogenic program. Consistent with our prior work, we show here that myoblasts overexpressing GRβ have a marked reduction in PTEN transcript levels. PTEN knockdown in rat myoblasts increased myosin heavy chain expression threefold in early stage myotubes and nearly doubled the differentiation rate [26]. Our present findings are in support of the studies mentioned above, and the first to show that the overexpression of GRβ reduced PTEN mRNA levels, increased the expression of the muscle regulatory factors *MyoD* and *myogenin*, and enhanced myotube formation. Collectively, these data support pro-myogenic and insulin-responsive properties of GRβ that may help preserve muscle mass in response to the negative affects of GC on skeletal muscle metabolism.

It is well documented that GC-induced GR activity induces muscle wasting by stimulating the proteolytic activity of the ubiquitin-proteasome pathway. The working paradigm suggests that in response to elevated endogenous or exogenous GCs, Foxo transcription factors increase MAFbx and MuRF1 expression to stimulate ubiquitin activity in skeletal muscle [28,29,34]. In this study, we reported that overexpression of GRβ suppressed Foxo3a expression and Dex-induced mRNA changes in MAFbx and MuRF1. While the mechanism(s) requires further investigation, our data suggests that GRβ can impair GRα mediated atrogenic signaling by suppressing a member of the Foxo family known to enhance proteolytic activity.

Waddell *et al.* [29] determined that GR and Foxo1 have independent response elements on the MuRF1 promoter, and act synergistically to induce muscle atrophy. However, while Dex increased the expression of MuRF1 and MAFbx *in vivo* [34], muscle mass was spared only in MuRF1 and not MAFbx knockout mice following denervation [32]. Interestingly, despite muscle sparing following denervation, MuRF1 knockout out mice displayed increases in proteasome activity [47] due to elevated expression of other regulators of proteasome-mediate ubiquitination. In contrast, MAFbx but not MuRF1 gene expression increased in response to Dex or corticosterone in C_2_C_12_ myotubes [31]. Here, we showed an increase in both atrogenes following 24 h of dexamethasone exposure. Furthermore, dexamethasone and corticosterone increased protein degradation in C_2_C_12_ myotubes with no effect of protein synthesis rates [31]. Lastly, in L6 muscle cells and rats, MAFbx was shown to be under the control of Foxo3a [28]. Through targeted siRNA knockdown of the insulin receptor substrate proteins and dexamethasone treatments *in vivo*, MAFbx expression was regulated by a suppression of the canonical insulin signaling pathway (IRS1/PI3K/AKT) and an increase in IRS2/MEK/ERK signaling [28]. These prior findings provide critical signaling networks in the regulation of atrogene expression that will be the target of future work in identifying the mechanisms by which overexpression of GRβ reduces MAFbx and MuRF1 Dex responsiveness.

The degradation of muscle regulatory factors is one mechanism by which MAFbx and MuRF1 induce destabilization of skeletal muscle and loss of muscle mass. Here, we show that overexpression of GRβ increased MyoD and myogenin gene expression, two muscle regulatory factors necessary for skeletal muscle development and regeneration [23]. Through protein-protein interactions, MAFbx mediates MyoD [48] and myogenin [49] protein ubiquitination. In response to dexamethasone, myogenin shows a decrease in protein content by 12 h or exposure, and almost complete loss by 24 h, corresponding to a peak in MAFbx expression [49]. In the current study, we show a significant reduction in MAFbx mRNA in GRβOE compared to vector cells when treated with Dex. Together with a ~2.5-fold increase in myogenin and ~1.8-fold increase in MyoD gene expression, the overexpression of GRβ in muscle cells may preserve skeletal muscle mass in the presence of GC, the true impact of which requires a skeletal muscle-specific *in vivo* approach to evaluate.

## 4. Materials and Methods

### 4.1. Cell Culture

All cells proliferated in HyClone DMEM (Fisher Scientific, Pittsburg, PA, USA) containing 10% FBS (Denville Scientific, Holliston, MA., USA) supplemented with 1% penicillin/streptomycin. (Alkali Scientific, Pompano Beach, FL, USA). Differentiation into myotubes was induced in cultures that reached ~90% confluence by switching to DMEM containing 2% horse serum (ATCC) and 1% penicillin/streptomycin. Dexamethasone (Calbiochem/EMD Millipore, Billerica, MA, USA) and insulin (Sigma Aldrich, St. Louis, MO, USA) treatments were for durations specified in the figure legends, at a concentration of 100nM (dexamethasone) and 100uM (insulin). For all experiments, cells were maintained in 5% CO_2_, 21% O_2_, and 37 °C.

### 4.2. Cell Lines

C_2_C_12_ mouse myoblasts were passaged in house. Cells passaged four times were used for experiments. To establish a C_2_C_12_ cell line with mouse GRβ stably overexpressed, mouse GRβ cDNA was ligated into the PacI/NotI sites of the pQXCIN vector that has an independent neomycin selection marker and transformed in DH5-α cells (Invitrogen/Fisher Scientific, Pittsburg, PA, USA). The construct was co-transfected together with vectors expressing gag-pol, REV, and VSV-G into 293FT cells (Invitrogen) to generate a third generation lentiviral construct. Transfection was achieved using GeneFect (Alkali Scientific, Pompano Beach, FL, USA) using 100 ng of total DNA per cm^2^ of the growth plate or well. The supernatants were harvested, and the cell debris was removed by centrifugation at 2000× *g*. The supernatant was used to infect C_2_C_12_ cells after the addition of polybrene (5 ng/mL, Sigma Aldrich, St. Louis, MO, USA) to establish cell lines with stable overexpression of GRβ mRNA (GRβOE) or those expressing an empty vector. After 72 h, the cells were selected using 500 mg/mL G418 [20].

### 4.3. Proliferation Assays

Vector and GRβOE cells were plated in 12-well plates in DMEM containing (1 × 10^4^ cells per well). The growth rate was determined as a function of time for 0–4 days of proliferation. Cell proliferation was determined by a calorimetric assay using MTT (3-(4,5-dimethylthiazol-2-yl)-2,5-dipheyltetrazoline bromide) as previously described [20].

*Quantitative Real-Time PCR Analysis.* Total RNA was extracted from cells using the 5-Prime PerfectPure RNA Cell Kit (Fisher Scientific, Pittsburg, PA, USA) and quantified using the NanoDrop 2000 spectrophotometer (Fisher Scientific, Pittsburg, PA, USA). cDNA was synthesized using the High Capacity cDNA Reverse Transcription Kit (Applied Biosystems). Real-time PCR amplification of the cDNA was performed using TrueAmp SYBR Green qPCR SuperMix (Smart Bioscience, Philadelphia, PA, USA) with a Step One Plus real-time PCR system (Applied Biosystems/Fisher Scientific, Pittsburg, PA, USA). Changes in gene expression were determined using the quantitative ΔΔC*t* method and normalized to GAPDH. A list of primer sequences is in Table 1.

### 4.4. Western Blot Analysis

Cells were harvested and lysed in a RIPA lysis buffer containing Holt protease and phosphatase inhibitors (Pierce/Fisher Scientific, Pittsburg, PA, USA). Protein content was determined using the BCA method (Pierce).

Western blot analysis was performed as previously described [50]. Briefly, 30 μg of protein was resolved by SDS-PAGE and transferred to Immobilon-FL membranes. Membranes were blocked at room temperature for 1 h with tris-buffered saline (TBS)/5% BSA, followed by two washes with TBS/0.1% tween 20 (TBS-T). Membranes were incubated overnight at 4 °C with FiGR antibody for total GR (Santa Cruz Biotechnology, Dallas, TX, USA, and rMGRβ antibody for mGRβ at a dilution of 1:1000 in TBS-T (the antibody was made as previously described [9]. Additionally, membranes were probed with β-actin (Sigma-Aldrich) at a dilution of 1:10,000 in TBS-T for two hours at 4 °C. After two washes in TBS-T, membranes were incubated with infrared anti-rabbit (IRDye 680) or anti-mouse (IRDye 800) secondary antibodies (LI-COR Biosciences, Lincoln, NE, USA) (1:15,000 dilution in a blocking buffer) for two hours at room temperature. Following two washes with TBS-T and one wash with TBS, immunoreactivity was visualized and quantified using the Odyssey Infrared Scanning system (LI-COR Biosciences).

### 4.5. Myosin Heavy Chain Immunofluorescence

Following four days of differentiation, plates were washed with PBS and fixed with cold 70% methanol/30% acetone for 10 min at room temperature. Myosin heavy chain staining was performed as previously described [50]. Cells were permeabilized with 0.05% triton-x 100 and blocked for 30 min at room temperature. Wells were incubated with anti-sarcomeric myosin heavy chain (MHC) MF20 (developed by Donald A. Fischman and obtained from the Developmental Studies Hybridoma Bank, The University of Iowa, Department of Biology, Iowa City, IA, USA) diluted 1:20 in a blocking buffer for two hours at room temperature. Wells were washed and incubated with goat anti-mouse FITC secondary antibody (Invitrogen) diluted 1:200 in PBS for 30 min at room temperature. Cover slips were mounted with Vector Shield containing 4′,6-diamidino-2-phenylindole (DAPI) (Vector Labs, Burlingame, CA, USA).

### 4.6. Quantification of Fusion and Myotube Formation

Blinded investigators took all pictures and performed quantification after demonstrating proficiency using practice images. MHC positive (MHC+) cells were viewed at 10× magnification. A myotube was considered a MHC+ cell with two or more nuclei. Five to seven fields were viewed per well in a predetermined manner, starting from the center of the well; the stage was moved two complete fields to the right (field 1), two fields up (field 2), two fields to the left (field 3), two fields to the left (field 4), two fields to the down (field 5), two fields down (field 6) and two fields to the right (field 7). For each field, one picture of MHC+ cells and one picture of Hoechst-labeled nuclei were taken and merged. Quantification was performed on printed merged images of each field. To determine how many total nuclei were within myotubes, all of the nuclei within MHC+ cells that contained two or more nuclei were counted. The number of nuclei per myotube was determined by dividing the number of myotubes in each image by the total number of nuclei counted within myotubes. The fusion index was determined using the following formula: (nuclei within myotubes/total nuclei × 100.

### 4.7. Statistical Analysis

Data was analyzed using Prism 6 software (GraphPad Software, San Diego, CA, USA). A one-way analysis of variance (ANOVA) combined with Tukey’s post-test was used to determine changes in GR protein expression in response to insulin and dexamethasone and changes in MyoD, myogenin, GRα and GRβ mRNA levels through four days of differentiation and the responsiveness of MAFbx and MuRF1 to dexamethasone in GRβOE cells. A two-way ANOVA with a Tukey’s post-test was used to determine difference in proliferation rates from the MTT assay. A student’s *t*-test was used to determine difference in gene expression in response to GRβ overexpression and parameters of fusion.

## 5. Conclusions

The present study is the first to characterize the effects of GRβ overexpression on the myogenic program in C2C12 muscle cells. While these data are informative, they prompt several pertinent questions open for future investigation. For example, GC-mediated MuRF1 expression causes the degradation of structural proteins in skeletal muscle [33,51,52,53]. As overexpression of GRβ reduced the Dex-mediated increase in MuRF1, it remains unknown how this affects the integrity of the myosin heavy chain isoforms and other structural proteins. Importantly, the present study justifies examining the response of GRβ to pharmacological, physical, and social mechanisms of increased GC exposure *in vivo*, and testing whether its overexpression is protective. As new evidence begins to emerge on the role of GRβ in skeletal muscle, we believe that this GR isoform may lead to the ability to target GC resistance to skeletal muscle and minimize proteolytic activity during treatment. Importantly, future studies should employ techniques that preserve the important therapeutic effects of GCs in other tissues, while preventing skeletal muscle catabolism, such as tissue-specific transgenic mouse models. Lastly, the role of GC resistance in metabolic disease should also be considered when designing future models [54].

## Figures and Tables

**Table 1:** Real-time PCR Primer Sequences.

Gene	Sequence 5’ to 3’
*GAPDH*	Forward: ATGTTTGTGATGGGTGTGAA
	Reverse: ATGCCAAAGTTGTCATGGAT
*MyoD*	Forward: TACCCAAGGTGGAGATCCTG
	Reverse: GCATCTGAGTCGCCACTGTA
*Myogenin*	Forward: CGCGATCTCCGCTACAGA
	Reverse: TGGGACCGAACTCCAGTG
*MAFbx*	Forward: CCAGGATCCGCAGCCCTCCA
	Reverse: ATGCGGCGCGTTGGGAAGAT
*MuRF1*	Forward: GGGGGTCAGGGGACGAAGACA
	Reverse: TCTCGCCCACCTGCGTCACA
*GRα*	Forward: AAAGAGCTAGGAAAAGCCATTGTC
	Reverse: CTGTCTTTGGGCTTTTGAGATAGG
*GRβ*	Forward: CAATCATGTTGCAGCAATTCACT
	Reverse: CCCCATAAAAATCTAGGGCCTCT
*Foxo3a*	Forward: GAGCTGGAGCTCGAACCTT
	Reverse: CTTGGGCTCTTGCTCTCTCC

GRα: glucocorticoid receptor α; GRβ: glucocorticoid receptor β; MAFbx: muscle atrophy F-box; MuRF1: muscle ring finger 1; GAPDH: glyceraldehyde-3-phosphate dehydrogenase; Foxo3a: forkhead box O3.

## Abbreviations

GC	glucocorticoid
GRE	glucocorticoid response elements
GR	glucocorticoid receptor
GRα	glucocorticoid receptor α
GRβ	glucocorticoid receptor β
GRβOE	glucocorticoid receptor β overexpressing cells
MAFbx	muscle atrophy F-box
MuRF1	muscle ring finger 1
FOXO	forkhead box O
PTEN	phosphatase and tensin homolog deleted on chromosome 10
IRS1 and 2	insulin receptor substrate 1 and 2
PI3K	phosphoinositide 3-kinase
